# 3D-printed mouthpiece adapter for sampling exhaled breath in medical applications

**DOI:** 10.1186/s41205-022-00150-y

**Published:** 2022-08-09

**Authors:** Y Lan Pham, Jonathan Beauchamp, Alexander Clement, Felix Wiegandt, Olaf Holz

**Affiliations:** 1 Fraunhofer Institute for Process Engineering and Packaging IVV, Giggenhauser Straße 35, 85354 Freising, Germany; 2 Department of Chemistry and Pharmacy, Chair of Aroma and Smell Research, Friedrich-Alexander-Universität Erlangen-Nürnberg, Henkestraße 9, 91054 Erlangen, Germany; 3 Fraunhofer Institute for Toxicology and Experimental Medicine ITEM, Feodor-Lynen-Str. 15, 30625 Hannover, Germany; 4 Member of the German Centre of Lung Research DZL (BREATH), Hannover, Germany

**Keywords:** Prototyping, Sampling Interface, Resin-printed Device, Stereolithography, Breath Analysis, Spirometry

## Abstract

The growing use of 3D printing in the biomedical sciences demonstrates its utility for a wide range of research and healthcare applications, including its potential implementation in the discipline of breath analysis to overcome current limitations and substantial costs of commercial breath sampling interfaces. This technical note reports on the design and construction of a 3D-printed mouthpiece adapter for sampling exhaled breath using the commercial respiration collector for in-vitro analysis (ReCIVA) device. The paper presents the design and digital workflow transition of the adapter and its fabrication from three commercial resins (Surgical Guide, Tough v5, and BioMed Clear) using a Formlabs Form 3B stereolithography (SLA) printer. The use of the mouthpiece adapter in conjunction with a pulmonary function filter is appraised in comparison to the conventional commercial silicon facemask sampling interface. Besides its lower cost – investment cost of the printing equipment notwithstanding – the 3D-printed adapter has several benefits, including ensuring breath sampling via the mouth, reducing the likelihood of direct contact of the patient with the breath sampling tubes, and being autoclaveable to enable the repeated use of a single adapter, thereby reducing waste and associated environmental burden compared to current one-way disposable facemasks. The novel adapter for breath sampling presented in this technical note represents an additional field of application for 3D printing that further demonstrates its widespread applicability in biomedicine.

**Supplementary Information:**

The online version contains supplementary material available at 10.1186/s41205-022-00150-y.

## Introduction

3D printing for healthcare applications has experienced a surge in interest in recent years due to advancements in the technical performances of printers, the emergence of suitable printing composite materials, and the widespread availability of low-cost printing devices [1, 2]. The rapid growth of 3D printing applications in the medical field is expected to revolutionise healthcare [3] and has been suggested to be of particular benefit in times of health crises when 3D printed materials can offer on-demand alternatives to medical parts and equipment that may be in short supply [4–7]. Prevalent biomedical applications include customised prosthetics [8, 9], implants [10], tissue and organ fabrication [11, 12], or anatomical models for medical training purposes [13–15], amongst others. Since their first reported use in medicine in the early 2000s [16, 17], 3D printers have evolved to become a reliable engineering tool in this field, providing the freedom to produce bespoke medical products and fittings with enhanced and cost-effective productivity [18].

A hitherto niche area within the expansive field of medical research is breath analysis, which has emerged in recent years as a promising approach to non-invasive disease diagnostics [19]. Exhaled breath contains a rich mixture of volatile chemical compounds [20], and the basic premise of breath analysis is to examine these constituents as a means to seek (early) indications of illness or infection. The benefits of breath analysis over conventional diagnostics using blood are its non-invasive (painless) sampling combined with rapid detection, offering the potential for widespread implementation in the areas of clinical diagnosis, routine screening, and therapeutic monitoring [21, 22].

In breath research, exhaled breath can be sampled for either on-line or off-line analysis. The former involves the direct connection of a breath sampling interface to the analyser to allow for immediate analysis of exhaled breath gas [23], whereas the latter involves the collection of a breath sample in a storage medium, which is then transferred to the analytical platform for subsequent analysis [21]; the respective benefits and drawbacks of these two approaches are discussed in the scientific literature [24, 25]. The most prevalent approach to sampling exhaled breath for breath gas analysis is the off-line method.

Due to the broad nature of breath research, whereby a wide variety of analytical technologies are used to target an extensive range of compounds for a diverse assortment of illnesses, a standardised approach to sampling for off-line analysis does not exist [26]. Off-line sampling methods include the collection of breath into inflatable bags, or its collection and pre-concentration onto adsorbent materials, allowing for trace level detection of breath-borne volatiles [27]. The most common approach utilises thermal desorption (TD) tubes – i.e., hollow tubes containing an adsorbent material with a high affinity to organic compounds – for sample collection, which are then interfaced with a gas chromatography-mass spectrometry (GC-MS) analyser, whereby the tubes are heated to liberate the trapped compounds into the analyser, i.e., thermal desorption, to enable their subsequent detection [28].

Sampling systems to collect breath volatiles on TD tubes are often custom built by individual research groups, but commercial devices exist, such as the Respiration Collector for In-Vitro Analysis (ReCIVA) breath sampler (Owlstone Medical Ltd., Cambridge, UK), which captures end-tidal breath directly onto TD tubes using a capnography-driven sampling protocol and associated control software [29]. The breath collector is conventionally equipped with a silicon facemask – similar to an oxygen mask used for ventilation in the clinical setting – that offers a snug fit over the mouth and nose of the participant and allows for rebreathing and sample collection [29–32]. Four holes at the bottom of the mask allow for insertion of the TD tubes, which protrude into the inside of the mask, located directly in-line with the wearer’s breathing flow. An integrated sterility filter at the downstream end of the mask ensures that the ReCIVA device is shielded from exposure to aerosols in the exhalation flow, thereby reducing the risk of cross-contamination when using the same device successively for different patients.

Despite some benefits of convenience, this facemask has several drawbacks. Foremost, its design does not permit the control of breathing solely via the mouth, but rather allows for mixed nasal-oral breathing; in breath analysis, the breathing route can directly impact the type and abundance of volatiles, since some compounds have production sites in the nasal cavity, whereas others originate from the oral cavity [33–35], thus a consistent exhalation route is imperative. Another limitation of the mask is the very close proximity of the TD tubes to the wearer’s face (mouth and nose), resulting in a high likelihood of direct physical contact to the non-sterile tubes (depending on anatomy) with an associated risk of infection to the potentially vulnerable patient, as well as a propensity to contaminate the tubes with aerosols present in exhaled breath (see Figure S1). Finally, the masks exhibit a short shelf-life of sterility and single-use performance, which are associated with considerable cost and waste for large cohort studies.

To address these shortcomings, an alternative mouthpiece adapter for connection to the ReCIVA device was designed for 3D stereolithography (SLA) printing to replace the facemask as a cost-effective and reusable alternative that ensures controlled exhalation through the mouth and a lesser likelihood of direct contact between the patient and the sampling tubes, thus reducing the risk of infection and cross-contamination between patients.

This technical note presents details of the design and construction of this inexpensive 3D-printed interface, with a view to making it available for wider use and potential adaptation and adoption by the breath research community in combination with the ReCIVA breath collection device as a viable alternative to the silicon facemask.

## Methods

**Fig. 1:**
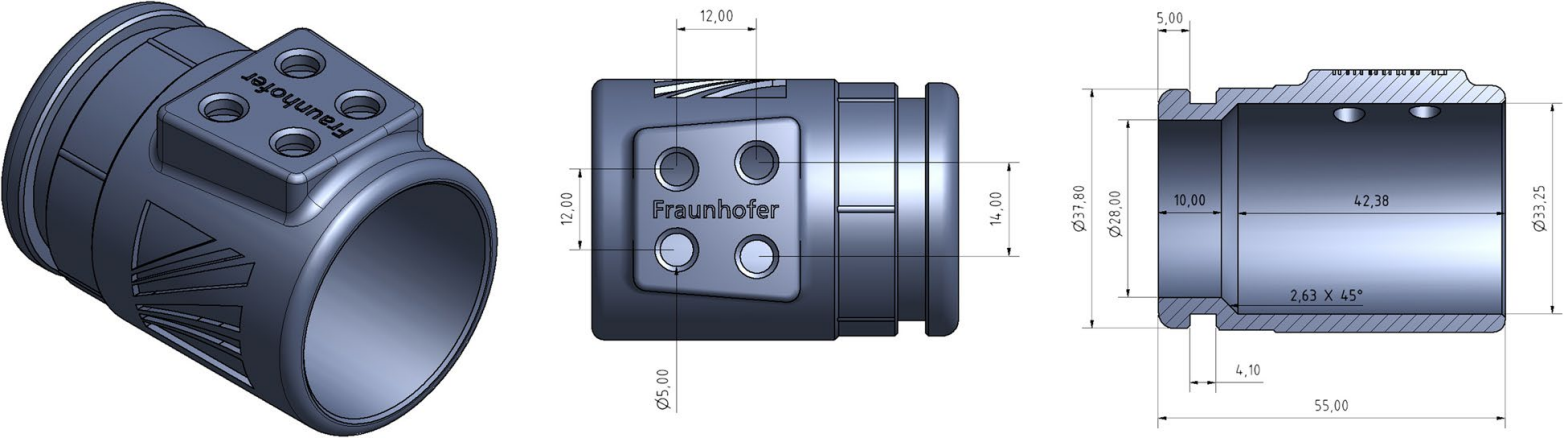
Model of the breath sampling adapter, viewed from three perspectives. Left: oblique view, showing the connection to the sampling device (facing rear-left) and the underside of the adapter (top/centre); centre: bottom view, showing the underside of the adapter and the connection orifices for the four adsorbent tubes in trapezoid formation; and right: cross-sectional planar view, showing the side of the adapter (the adapter connects to the breath sampling device on the left-hand (rear) end; a pulmonary function filter is attached at the right-hand (front) end); the base of the adapter, containing the connection orifices for the adsorption tubes, is depicted at the top. All dimensions are in mm

**Fig. 2:**
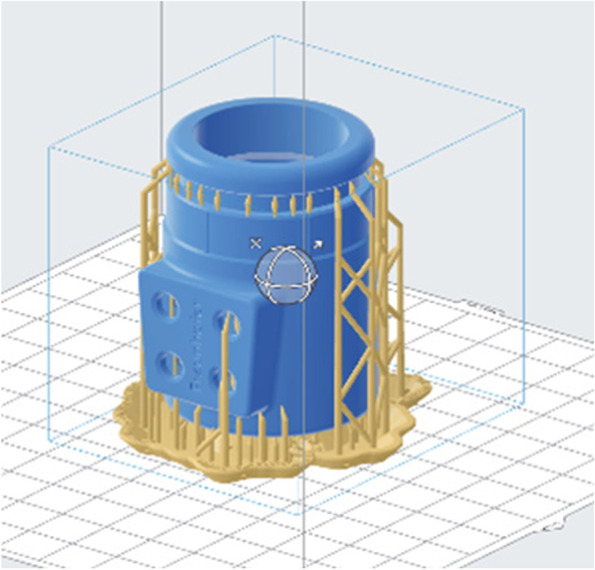
Model of the adapter (blue) for 3D printing in an upright position, displaying the vertical support structures (ochre colour)

**Fig. 3:**
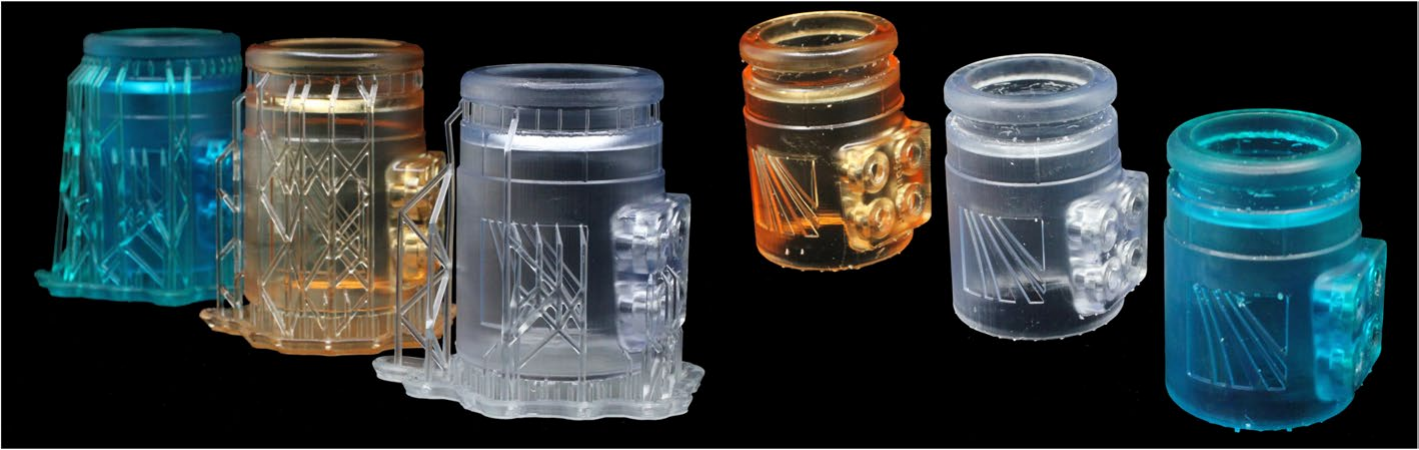
Final adapter models pictured with (left) and without (right) support structures, printed from Tough v5 (blue), Surgical Guide (orange) and BioMed Clear (translucent) resins

A routine workflow for model design and 3D-printing was undertaken, namely to establish an *in silico* three-dimensional model of the object for subsequent 3D printing into its physical representation. In a first step, a 3D prototype model was created using Autodesk Inventor Professional computer-aided design (CAD) software (Inventor 2015 Build: 159, Release 2015 RTM., Autodesk Inc., CA, USA). The 3D model was exported to Standard Tessellation Language (STL) format, which contains mesh coordinates of 3D models (depicted in Fig. 1). Next, the Formlabs PreForm software (v. 3.19.1., Formlabs Inc., Somerville, MA, USA) was used to convert the STL file into a 3D printable format (.form), which includes printing information, such as layer thickness, printing material, orientation, and support structures (see Figs. 2 and 3) in order to facilitate printing. Finally, the ensuing files were exported for the individual resin materials and transferred to the printer for 3D printing.

### CAD model and 3D-printing of the adapter

**Fig. 4:**
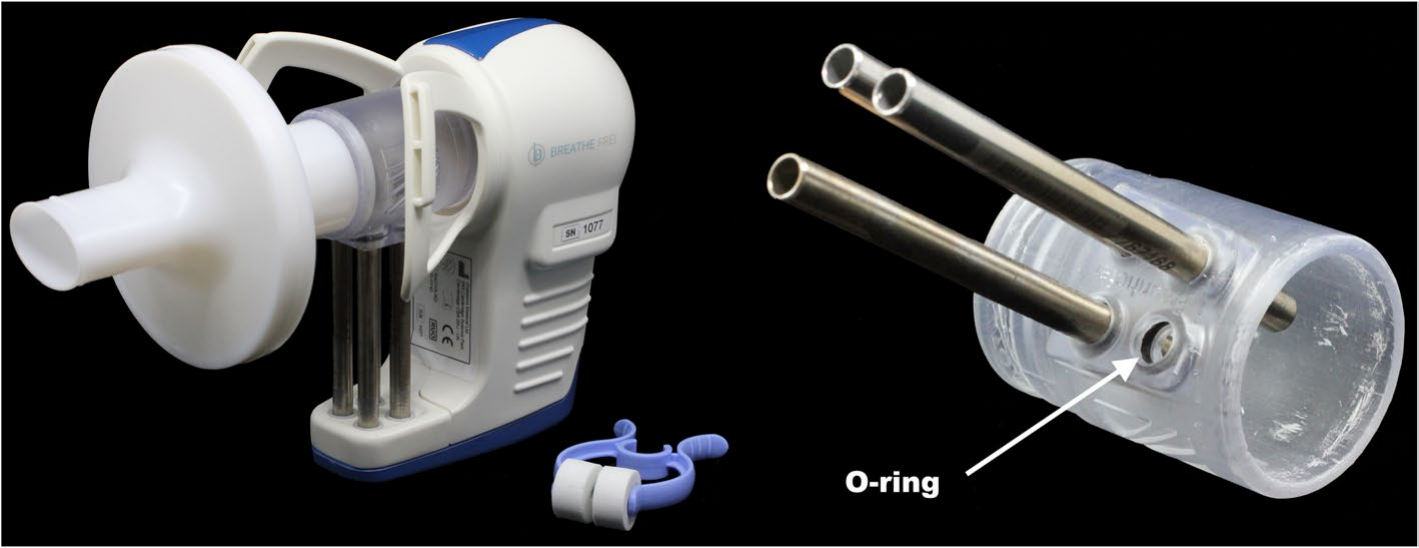
Left: Assembled breath sampling device comprising the ReCIVA breath sampler (right), a 3D-printed mouthpiece adapter (an adapter made from BioMed Clear resin is depicted; centre), a disposable pulmonary function filter (left), and thermal desorption tubes to sample breath (bottom-centre). The nose clip depicted is used as an adjunct during breath sampling to ensure mouth breathing. Right: Close-up of the underside of the adapter with three inserted thermal desorption tubes and one open tube orifice fitted with an O-ring

The initial concept and development of the breath sampling adapter was motivated by a need to overcome the limitations of the current commercial silicon facemasks. This modification centres on an adapter that can be mounted directly to the ReCIVA breath sampling device and enables the simultaneous collection of volatile constituent of breath onto up to four independent TD tubes by exhaling through the hollow cylindrical adapter. The adapter can be paired with a commercially available pulmonary function filter to reduce the risk of cross-infection between participants (see Fig. 4) and the adapter itself can be treated for reuse, e.g., by autoclaving.

The core design of the adapter is a cylindrical tube of 55 mm length and 39 mm outer diameter. Its inner diameter for most of its length is 33 mm, but with an inner lip of 28 mm at the downstream end where it connects to the sampling device, allowing it to click firmly into place (see Fig. 1). A trapezoid-shaped protrusion along the axial direction and located at the back half of the adapter allows for connection of the TD tubes. To enable their connection to the adapter, the protrusion houses four orifices of 5.0 mm diameter – subsequently enlarged post-printing to 6.3 mm (see later) – that are positioned in a slight trapezoid configuration. These holes are furnished with sunken rims to allow for the insertion of Viton O-rings (6.3 × 2.0 mm inner diameter × thickness) to provide a firm attachment of standard size TD tubes (0.25" × 3.50" outer diameter × length), as well as to ensure isolation of the tubes from ambient air exterior to the adapter (see Fig. 4). The aforementioned tapered rim at the rear end of the adapter allows for its firm connection to the ReCIVA device.

All models were printed using a Form 3B printer (or Form 2 printer for earlier prototypes; not depicted here) (Formlabs Inc.) and a selection of different resins, namely Tough v5, Surgical Guide and BioMed Clear (Formlabs Inc.). The use of three different resins was to explore their different properties and related suitability for breath sampling purposes. Printing proceeded using a 100 μm layer thickness build at a pre-programmed printing temperature of 35 °C. The support structures were created automatically by the printer. After printing, the uncured models underwent post-processing. This comprised: washing (whilst still attached to the print bed) for 20 min in >95% isopropyl alcohol (IPA) in a washing station (Form Wash, Formlabs Inc.) to ensure removal of any residual uncured resin; physical detachment from the print bed; post-curing under ultraviolet (UV) light in a cure station (Form Cure, Formlabs Inc.) in accordance with the manufacturer guidelines specific to each resin material; and finally, manual removal of the support structures using cutting pliers. Additionally, any residual protrusions or rough areas remaining on the printed surfaces after removal of the support structures were mechanically removed by abrasion (fine sandpaper) to achieve a smooth surface. The final, printed adapters for all three resins are depicted in Fig. 3, with and without supporting structures.

### Adapter use for breath sampling

Before the newly printed – or, subsequently, used – adapters may be used for their intended purpose, they must be treated to ensure cleanliness and sterility. One approach to achieve this is sterilisation by autoclaving (steaming). This process was undertaken and examined for the printed adapters, in order to assess their resilience to autoclaving and their performance after this treatment. Autoclaving proceeded by wrapping an individual adapter in aluminium foil (after removal of the O-rings) and applying steam sterilisation at 121 °C for 30 min using a benchtop autoclave (HMC 300 MBF; HMC Europe GmbH, Tüßling, Germany), followed by a 30 min drying phase. After this treatment, the adapters may be stored under sterile conditions (in packaging pouches) until their use for breath sampling. Alternative sterilisation methods include treatment with ethylene oxide (ETO) or plasma sterilisation. Autoclaving was used in the present study following manufacturer recommendations of steam sterilisation for these resins.

To assemble the breath sampling device, O-rings are first placed in the four orifices of the adapter, then (up to) four TD tubes are inserted into these orifices, ensuring a snug fit (see Fig. 4). The other ends of the tubes are then pushed down into the four hollow insets at the bottom of the ReCIVA device. The ReCIVA device allows for sampling onto any one or all of the four tubes; if exhaled breath is to be sampled on less than four tubes, empty tubes or dummy rods of identical dimensions to the tubes can be inserted in the unused positions to maintain structural rigidity. After the tubes have been attached in the described manner, the front end of the adapter can be fitted with a pulmonary function filter, after which the grooved (rear) end is connected to the socket of the ReCIVA device, as depicted in Fig. 4.

## Results and Discussion

### CAD adapter design and 3D-printed models

The choice of resins used to produce the prototype adapters described herein was made according to their expected suitability for the intended purpose, based on the material datasheet specifications of the manufacturer. Tough v5 resin (discontinued, but available at the time of writing in modified composition as Tough 2000) is described as exhibiting a particularly sturdy structure, thus promising long-term durability. By comparison, Surgical Guide resin is promoted as non-cytotoxic, non-irritative and non-sensitising according to the ISO 10993-1:2018 standard, thereby representing a particularly attractive material for the adapter because of its intended use with human subjects. Lastly, BioMed Clear resin has passed biocompatibility requirements of the ISO 10993-1:2018, ISO 7405:2018 and ISO 18562-1:2017 standards, thus surpasses the properties of Surgical Guide resin by purportedly exhibiting no emissions of particulates, volatile organic compounds, or hazardous water-soluble substances. This latter aspect is essential in applications with direct contact to human subjects, and especially so in breath analysis to avoid inhalation of compounds or particulate matter on the one hand, and sampling of compound emissions as potential confounders on the other. An appraisal of the emissions is beyond the scope of this technical note but is currently under investigation for future dissemination.

Support structures of the printed model had different touchpoint sizes that ranged from 0.3 mm to 0.7 mm, depending on the resin used for printing. Trial printing runs for different orientations of the model yielded an upright position to be the most favourable for printing due to the absence of support structures on the inner surface of the adapter; in addition to the associated practical benefit of avoiding mechanical removal of these support structures at an inconvenient and poorly accessible location within the cylinder, the ensuing rough surface caused by the support structures are expected to have a higher adsorption potential of volatile compounds, which would be of detriment to the intended application of sampling breath volatiles. A drawback of this print orientation, however, was that the four orifices for insertion of the TD tubes were not uniformly round but rather slightly oval in shape. This was overcome by printing the orifices with a smaller diameter (5 mm), then using a bur to mechanically increase this diameter to accommodate the TD tubes (6.6 mm), thereby providing a firm attachment of the conventional 0.25" diameter TD tubes. (It might be noted that printing in horizontal orientation produces uniformly round holes, but presents the drawback described above in relation to the presence of supporting structures within the adapter tube; the vertical printing approach was deemed the most suitable to ensure precision for the adapter connection to the sampling system, and offered flexibility in achieving a snug fit for insertion of the four TD tubes.) In terms of yield, a standard 1 L resin tank can produce approximately 45 adapters, depending on the resin.

### Comparison of 3D-printed adapter with commercial facemask

Conventionally, the ReCIVA breath sampler is used with a silicon facemask to sample exhaled breath, which is delivered by the supplier (Owlstone Medical) under sterile conditions. The facemask offers the advantage of flexibility of the silicon that adapts to the contours of the wearer’s face, ensuring a snug fit. A further convenience of the facemask is its integrated sterility filter at the downstream end to reduce the risk of contamination of the ReCIVA device by the passage of exhaled breath during use. While the latter provides a degree of protection to the ReCIVA device that lessens the risk of cross-infection between patients, the close proximity of the wearer’s nose and mouth to the sampling tubes poses a risk of infection to the wearer, especially when breath is sampled from immunocompromised patients; the TD tubes cannot be treated sufficiently to eliminate this risk without compromising their sampling performance. The mouthpiece adapter presented here minimises this risk through use of a pulmonary function filter between the patient and the adapter, thus direct contact of the patient’s lips or nose with the TD tubes is prohibited. In practice, this configuration requires the additional use of a nose clip to ensure that the participant exhales exclusively through the mouth and inhales clean air provided by the CASPER Portable Air Supply (Owlstone Medical Ltd., Cambridge, UK) attached to the ReCIVA device, as is also routine configuration when using the facemask. However, the use of a nose clip can be considered an advantage, since sampling breath via the mouth is usually desirable in breath analysis, as discussed above.

In addition to the aforementioned limitations of the conventional facemask for sampling, the mask is relatively high in cost (ca. $ 25) for a single-use, disposable item and exhibits a limited sterility period of two weeks after purchase (treatment), as stipulated by the supplier. This represent a hindrance for its widespread cost-effective implementation for breath tests in routine analysis or large cohort clinical studies, and leads to high waste and an associated environmental burden. By comparison, the novel 3D-printed adapter presented herein exhibits properties that overcome some of the limitations of the facemasks. Considering expense, the cost of producing a single adapter is between $ 4-8, depending on the resin and based on current commercial prices (investment costs of the 3D printer and associated peripheral equipment notwithstanding). Taking into account the additional cost of the indispensable single-use pulmonary function filter for connection to the adapter (< $ 1), the printed adapter costs around one-third to one-fifth of the price of the conventional facemask. Notably, the possibility to sterilise the adapters for reuse multiple times further considerably reduces its cost and thereby increases its cost efficiency in studies with large cohorts and/or longitudinal sampling over a broad timespan. (Sterilisation of the silicon facemask is limited due to its in-built sterility filter, which is susceptible to deterioration during autoclaving or chemical treatment).

Another limitation consideration of the facemask in relation to the ensuing breath sample analysis is that the material itself is a potential source of confounding compounds and might equally act as a sink for volatile breath constituents through adsorption. Although this cannot be ruled out for the 3D-printed sampling adapter presented here, at least one of the resins (BioMed Clear) is specified to exhibit no volatile emissions, so its potential as a source of confounders is low.

A last aspect of the adapter to consider is its resilience to the autoclaving (sterilisation) process, specifically the potential changes in the physical properties of the material induced by the treatment, e.g., micro-fractures or apparent porosity. Although no explicit material properties assessments were undertaken in the present work, visual inspection yielded no visible changes after autoclaving, apart from a slight shift in hue for the Surgical Guide resin models, changing from orange to pale-yellow. Further, its firm connection to the ReCIVA device, as well as the insertion of the TD tubes, remained viable after autoclaving, indicating that any dimensional changes were minimal and did not compromise its further use.

Finally, it is worth noting that the adapter design presented in this technical note is versatile in that it can be modified depending on the intended application and equipment available for use as an adjunct to the ReCIVA device. As an example, the design can be adjusted to fit the requirements for sampling breath from children, or for use with an alternative/additional sterile filter located elsewhere within the sampling set-up. Notably, any modifications of a certified medical device conventionally render the certification invalid, thus this aspect must also be considered for the novel adapter presented here. However, the ReCIVA device is currently not certified as a medical device and is intended for research purposes only, thus the present modifications do not compromise this aspect of the sampler. Preliminary performance assessments indicate that exhaled breath sampled via the novel 3D-printed adapter achieves comparable levels of selected breath volatiles to samples collected with the commercial facemask [36]. Further, the ReCIVA system configuration presented in this technical note is currently in successful use in an ongoing clinical study, which reasserts its applicability in the intended setting [37].

## Conclusions

3D printing in medical science is an emerging practice that offers manifold benefits, primarily the cost-efficient fabrication and design flexibility of bespoke parts for specific and/or niche applications. The field of breath analysis represents a novel approach to disease diagnostics in the clinical environment and beyond [21]. Limited access to suitable tools for sampling breath, for example, due to budget constraints in procuring commercial adapters, poses a hindrance to advancement in this field of research. Consequently, a low-cost 3D-printed sampling adapter, as presented in this technical note, represents a feasible alternative to commercial products. The flexibility and versatility of 3D printers provide a freedom to operate and produce custom-made medical products and parts [38]. The availability of easily-accessible designs, such as via open-access platforms, is essential for transparency of the model and incremental improvements proposed by the user community, as well as for subsequent proliferation for widespread and cost-effective implementation of the designed model. This is especially relevant in periods of emergy, when shortages in regular supply chains might cause limitations in medical care. Overall, the use of 3D printing is set to surpass its current emphasis on prototyping to become more widely adopted for commercial use, especially as this technology continues to mature and as prototype models are sufficiently tested for biocompatibilty and safety for medical applications.

## Supplementary Information

**Additional file 1: Figure S1.** (left) Lateral view of the ReCIVA breath sampler equipped with a commercially available facemask. (right) Cross-sectional view of the silicon mask for an improved visibility of nose and mouth placement above the assembled thermal desorption tubes.

## Data Availability

All data and 3D printer files used in this technical note are available from the corresponding author upon reasonable request.

## Abbreviations

CAD: Computer-aided design
ETO: ethylene oxide
GC-MS: Gas chromatography-mass spectrometry
IPA: Isopropyl alcohol (isopropanol)
ReCIVA: Respiration collector for in-vitro analysis
SLA: Stereolithography
STL: Standard tessellation language
TD: Thermal desorption
UV: Ultraviolet (irradiation)
