# Supplementary material for: 3D-printed mouthpiece adapter for sampling exhaled breath in medical applications
Source: 3D Print Med. 2022 Aug 9;8:27. doi: 10.1186/s41205-022-00150-y (PMC9364600; doi:10.1186/s41205-022-00150-y)
Supplement: Supplementary file 1 — Additional file 1: Figure S1. (left) Lateral view of the ReCIVA breath sampler equipped with a commercially available facemask. (right) Cross-sectional view of the silicon mask for an improved visibility of nose and mouth placement above the assembled thermal desorption tubes. [file 41205_2022_150_MOESM1_ESM.pdf]

Supplementary file:
Paper: ***3D-printed mouthpiece adapter for sampling exhaled breath in medical applications***
Authors: Pham *et al.*

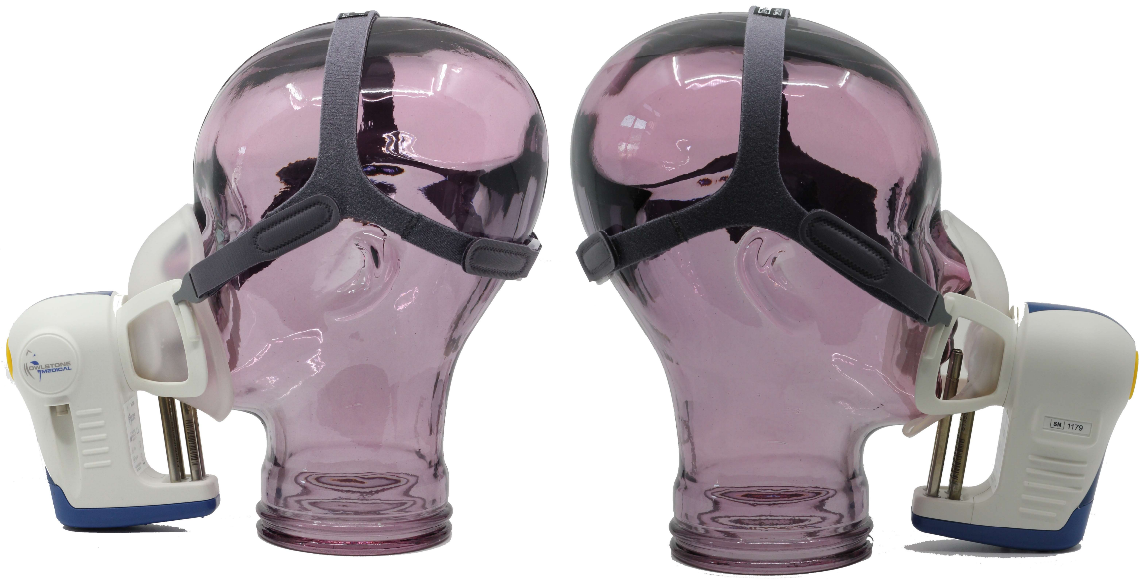

Figure S1: Left: Lateral view of the ReCIVA breath sampler equipped with a commercially available facemask and four adsorption tubes. Right: Cross-sectional cut-out view of the silicon mask for an improved visibility of nose and mouth position and close proximity to the assembled adsorption tubes.
